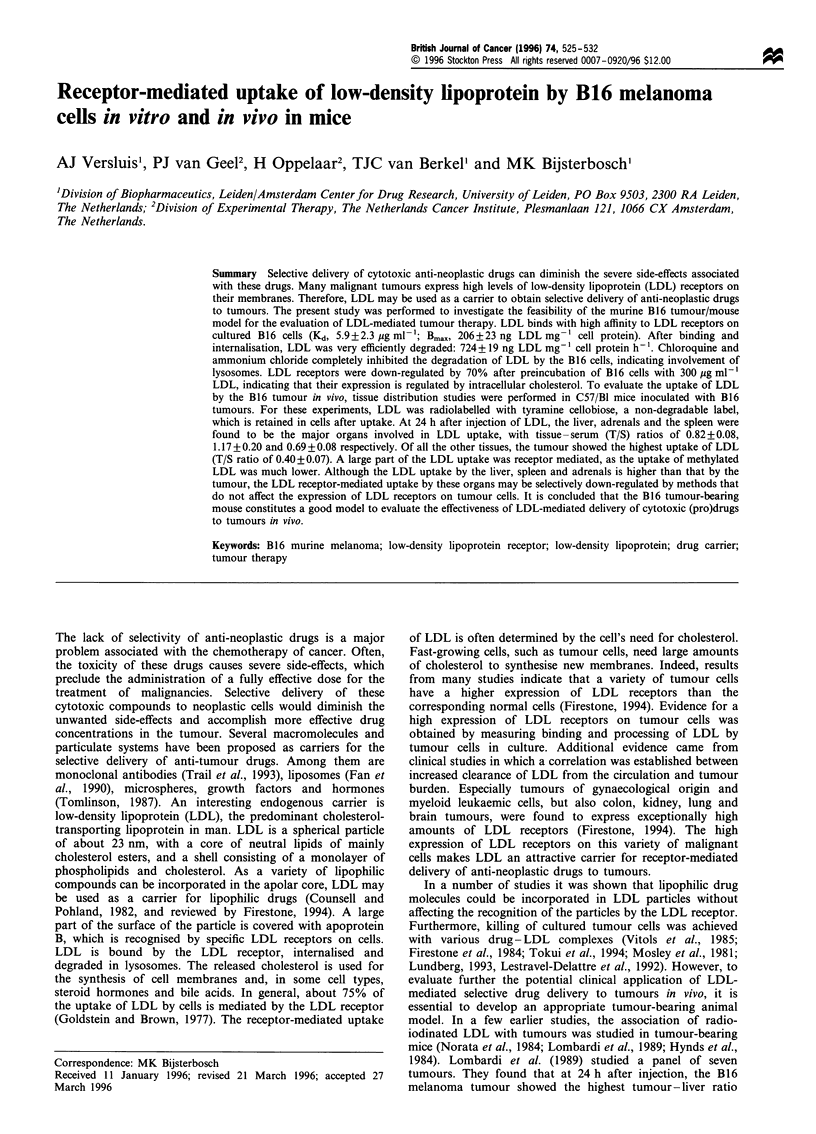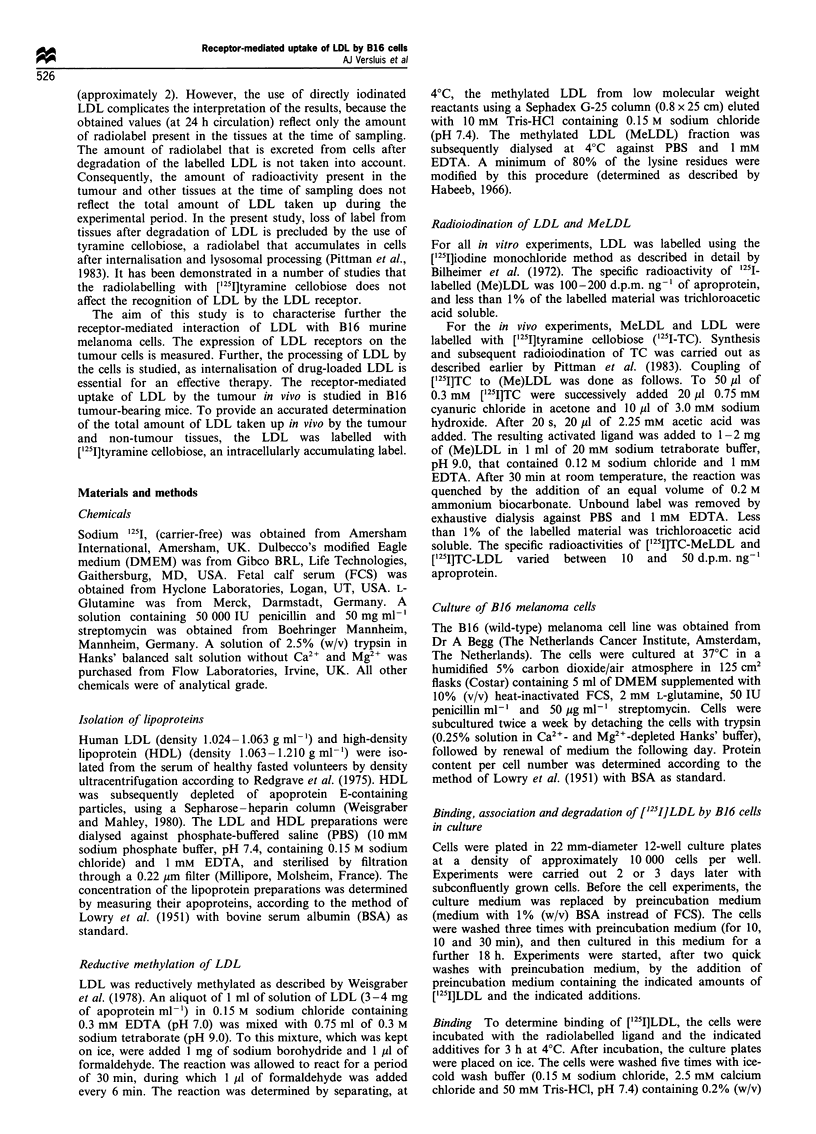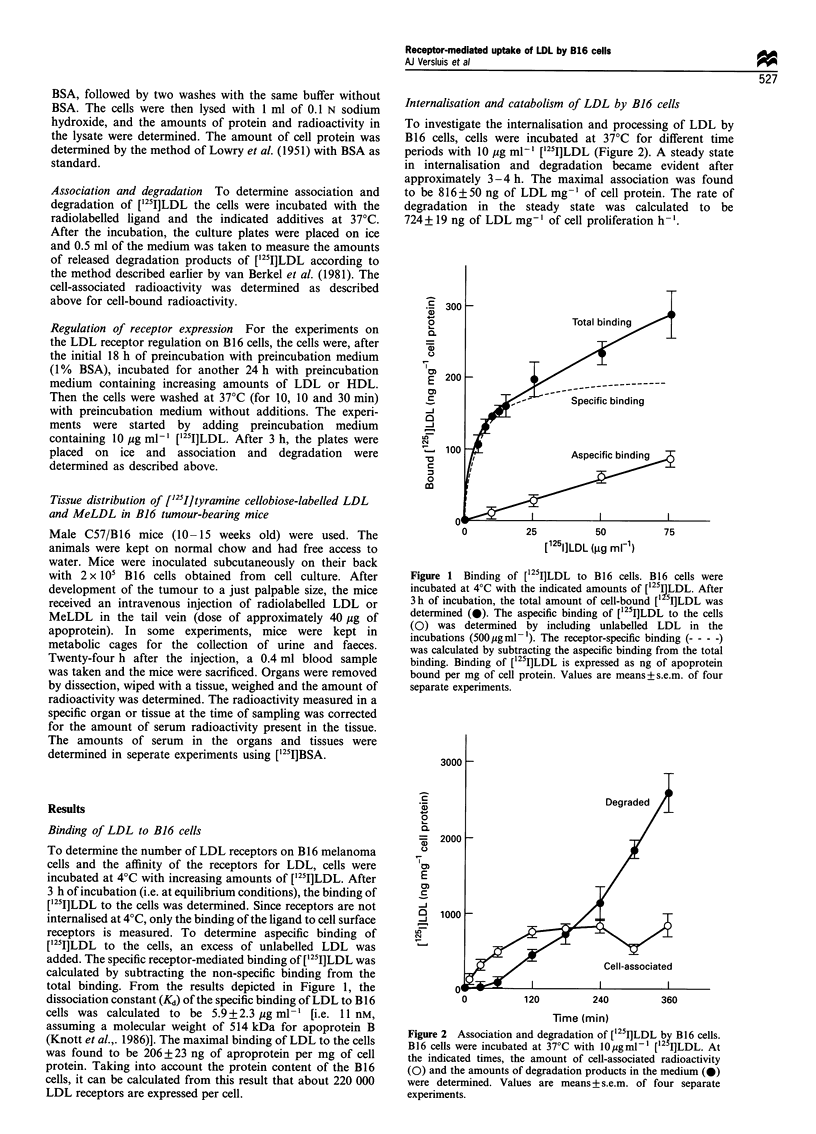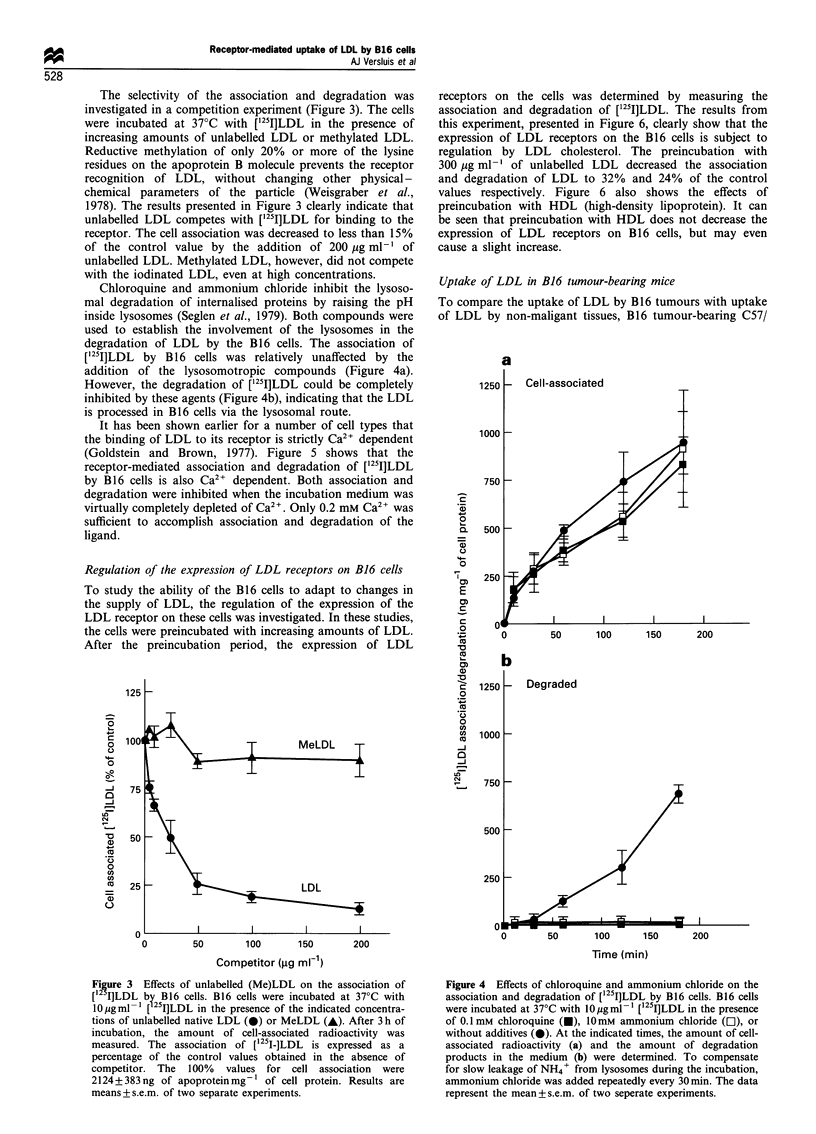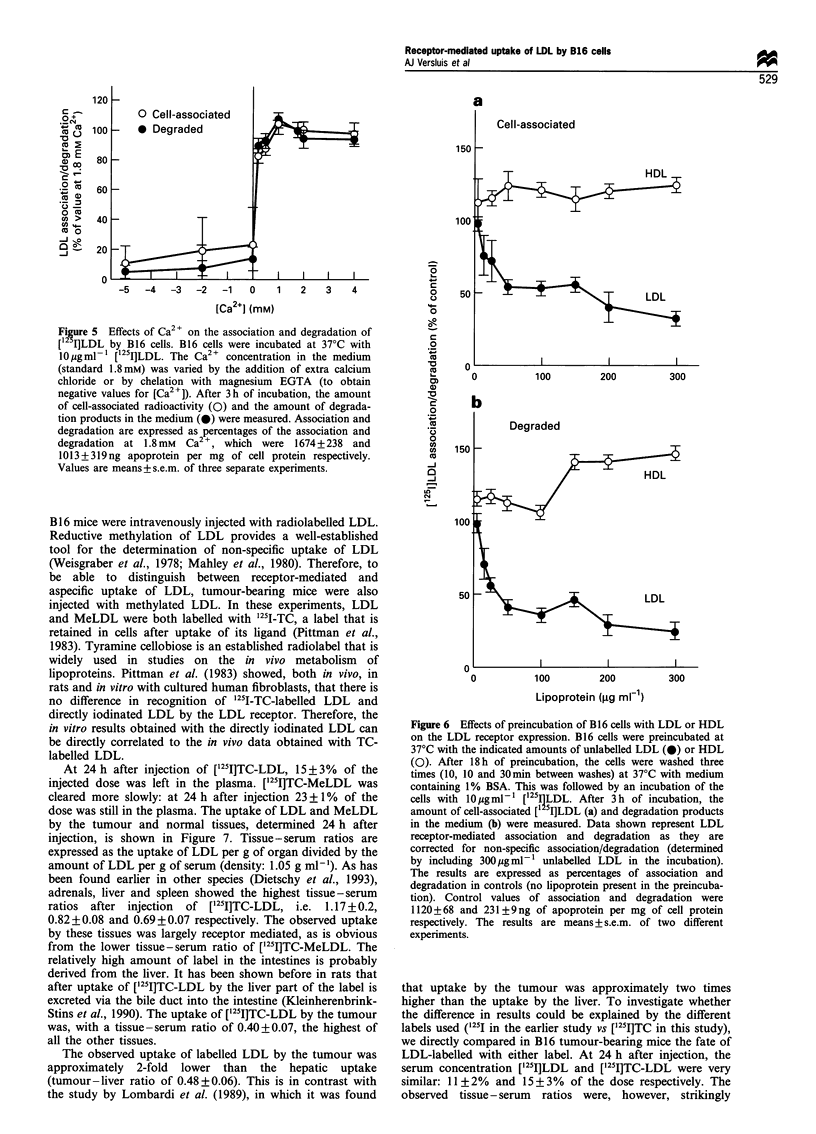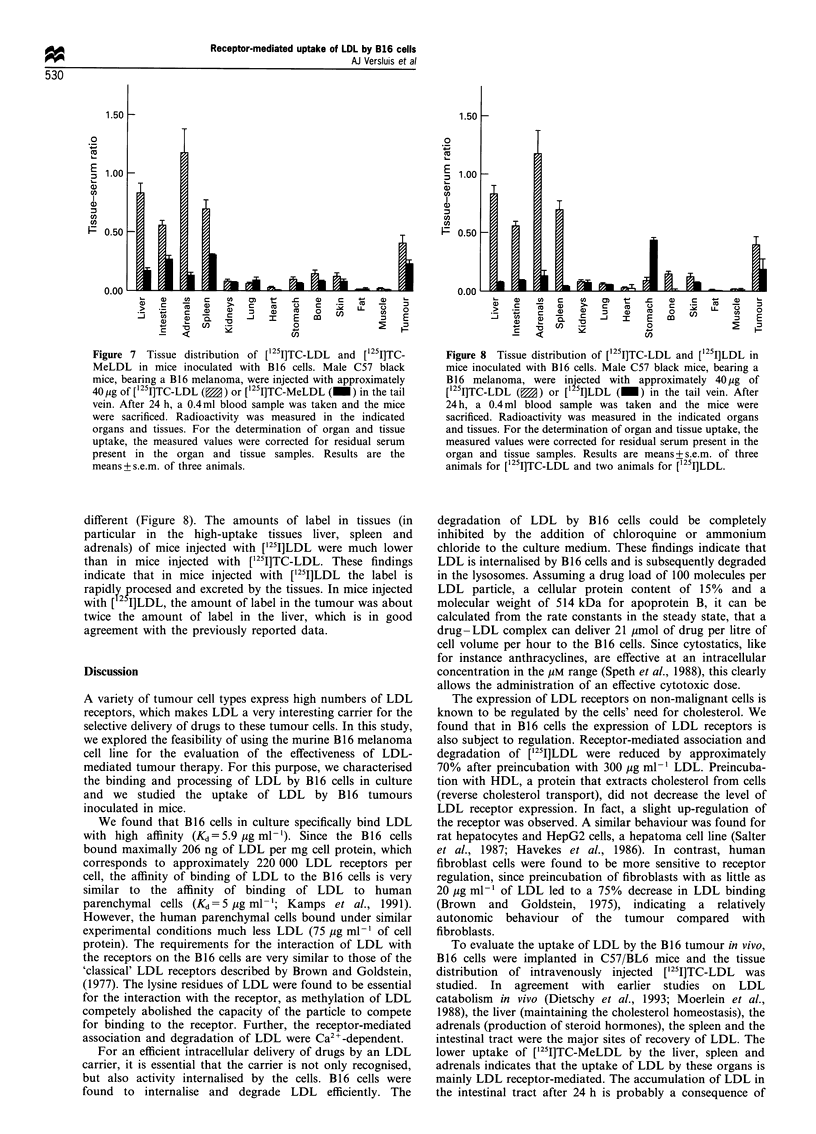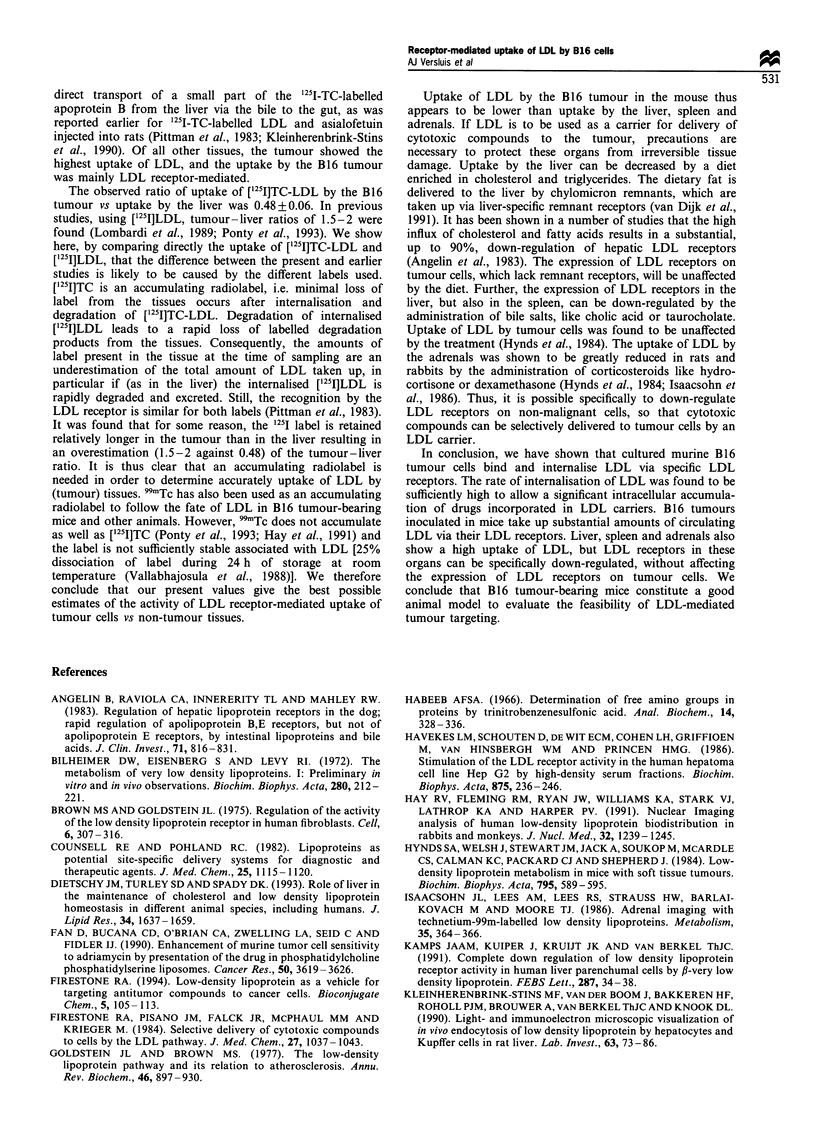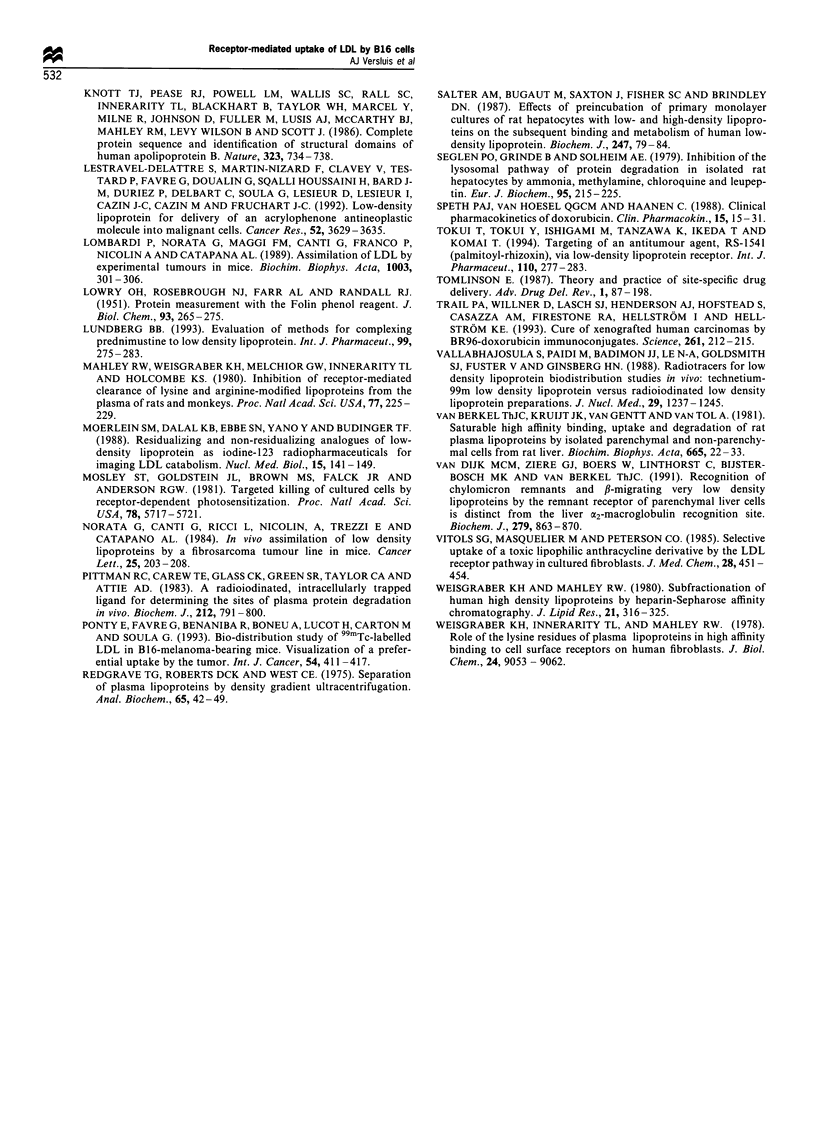# Receptor-mediated uptake of low-density lipoprotein by B16 melanoma cells in vitro and in vivo in mice.

**DOI:** 10.1038/bjc.1996.396

**Published:** 1996-08

**Authors:** A. J. Versluis, P. J. van Geel, H. Oppelaar, T. J. van Berkel, M. K. Bijsterbosch

**Affiliations:** Division of Biopharmaceutics, Leiden/Amsterdam Center for Drug Research, University of Leiden, The Netherlands.

## Abstract

Selective delivery of cytotoxic anti-neoplastic drugs can diminish the severe side-effects associated with these drugs. Many malignant tumours express high levels of low-density lipoprotein (LDL) receptors on their membranes. Therefore, LDL may be used as a carrier to obtain selective delivery of anti-neoplastic drugs to tumours. The present study was performed to investigate the feasibility of the murine B16 tumour/mouse model for the evaluation of LDL-mediated tumour therapy. LDL binds with high affinity to LDL receptors on cultured B16 cells (Kd, 5.9 +/- 2.3 micrograms ml-1; Bmax 206 +/- 23 ng LDL mg-1 cell protein). After binding and internalisation, LDL was very efficiently degraded: 724 +/- 19 ng LDL mg-1 cell protein h-1. Chloroquine and ammonium chloride completely inhibited the degradation of LDL by the B16 cells, indicating involvement of lysosomes. LDL receptors were down-regulated by 70% after preincubation of B16 cells with 300 micrograms ml-1 LDL, indicating that their expression is regulated by intracellular cholesterol. To evaluate the uptake of LDL by the B16 tumour in vivo, tissue distribution studies were performed in C57/B1 mice inoculated with B16 tumours. For these experiments, LDL was radiolabelled with tyramine cellobiose, a non-degradable label, which is retained in cells after uptake. At 24 h after injection of LDL, the liver, adrenals and the spleen were found to be the major organs involved in LDL uptake, with tissue-serum (T/S) ratios of 0.82 +/- 0.08, 1.17 +/- 0.20 and 0.69 +/- 0.08 respectively. Of all the other tissues, the tumour showed the highest uptake of LDL (T/S ratio of 0.40 +/- 0.07). A large part of the LDL uptake was receptor mediated, as the uptake of methylated LDL was much lower. Although the LDL uptake by the liver, spleen and adrenals is higher than that by the tumour, the LDL receptor-mediated uptake by these organs may be selectively down-regulated by methods that do not affect the expression of LDL receptors on tumour cells. It is concluded that the B16 tumour-bearing mouse constitutes a good model to evaluate the effectiveness of LDL-mediated delivery of cytotoxic (pro)drugs to tumours in vivo.


British Journal of Cancer (1996) 74, 525-532
© 1996 Stockton Press All rights reserved 0007-0920/96 $12.00

### Receptor-mediated uptake of low-density lipoprotein by B16 melanoma cells *in vitro* and *in vivo* in mice

AJ Versluis[1], PJ van Geel[2], H Oppelaar[2], TJC van Berkel[1] and MK Bijsterbosch[1]

[1]*Division of Biopharmaceutics, Leiden/Amsterdam Center for Drug Research, University of Leiden, PO Box 9503, 2300 RA Leiden, The Netherlands;* [2]*Division of Experimental Therapy, The Netherlands Cancer Institute, Plesmanlaan 121, 1066 CX Amsterdam, The Netherlands.*

**Summary** Selective delivery of cytotoxic anti-neoplastic drugs can diminish the severe side-effects associated with these drugs. Many malignant tumours express high levels of low-density lipoprotein (LDL) receptors on their membranes. Therefore, LDL may be used as a carrier to obtain selective delivery of anti-neoplastic drugs to tumours. The present study was performed to investigate the feasibility of the murine B16 tumour/mouse model for the evaluation of LDL-mediated tumour therapy. LDL binds with high affinity to LDL receptors on cultured B16 cells ($K_d$, $5.9 \pm 2.3$ μg $ml^{-1}$; $B_{max}$, $206 \pm 23$ ng LDL $mg^{-1}$ cell protein). After binding and internalisation, LDL was very efficiently degraded: $724 \pm 19$ ng LDL $mg^{-1}$ cell protein $h^{-1}$. Chloroquine and ammonium chloride completely inhibited the degradation of LDL by the B16 cells, indicating involvement of lysosomes. LDL receptors were down-regulated by 70% after preincubation of B16 cells with 300 μg $ml^{-1}$ LDL, indicating that their expression is regulated by intracellular cholesterol. To evaluate the uptake of LDL by the B16 tumour *in vivo*, tissue distribution studies were performed in C57/Bl mice inoculated with B16 tumours. For these experiments, LDL was radiolabelled with tyramine cellobiose, a non-degradable label, which is retained in cells after uptake. At 24 h after injection of LDL, the liver, adrenals and the spleen were found to be the major organs involved in LDL uptake, with tissue–serum (T/S) ratios of $0.82 \pm 0.08$, $1.17 \pm 0.20$ and $0.69 \pm 0.08$ respectively. Of all the other tissues, the tumour showed the highest uptake of LDL (T/S ratio of $0.40 \pm 0.07$). A large part of the LDL uptake was receptor mediated, as the uptake of methylated LDL was much lower. Although the LDL uptake by the liver, spleen and adrenals is higher than that by the tumour, the LDL receptor-mediated uptake by these organs may be selectively down-regulated by methods that do not affect the expression of LDL receptors on tumour cells. It is concluded that the B16 tumour-bearing mouse constitutes a good model to evaluate the effectiveness of LDL-mediated delivery of cytotoxic (pro)drugs to tumours *in vivo*.

**Keywords:** B16 murine melanoma; low-density lipoprotein receptor; low-density lipoprotein; drug carrier; tumour therapy

The lack of selectivity of anti-neoplastic drugs is a major problem associated with the chemotherapy of cancer. Often, the toxicity of these drugs causes severe side-effects, which preclude the administration of a fully effective dose for the treatment of malignancies. Selective delivery of these cytotoxic compounds to neoplastic cells would diminish the unwanted side-effects and accomplish more effective drug concentrations in the tumour. Several macromolecules and particulate systems have been proposed as carriers for the selective delivery of anti-tumour drugs. Among them are monoclonal antibodies (Trail *et al.*, 1993), liposomes (Fan *et al.*, 1990), microspheres, growth factors and hormones (Tomlinson, 1987). An interesting endogenous carrier is low-density lipoprotein (LDL), the predominant cholesterol-transporting lipoprotein in man. LDL is a spherical particle of about 23 nm, with a core of neutral lipids of mainly cholesterol esters, and a shell consisting of a monolayer of phospholipids and cholesterol. As a variety of lipophilic compounds can be incorporated in the apolar core, LDL may be used as a carrier for lipophilic drugs (Counsell and Pohland, 1982, and reviewed by Firestone, 1994). A large part of the surface of the particle is covered with apoprotein B, which is recognised by specific LDL receptors on cells. LDL is bound by the LDL receptor, internalised and degraded in lysosomes. The released cholesterol is used for the synthesis of cell membranes and, in some cell types, steroid hormones and bile acids. In general, about 75% of the uptake of LDL by cells is mediated by the LDL receptor (Goldstein and Brown, 1977). The receptor-mediated uptake of LDL is often determined by the cell's need for cholesterol. Fast-growing cells, such as tumour cells, need large amounts of cholesterol to synthesise new membranes. Indeed, results from many studies indicate that a variety of tumour cells have a higher expression of LDL receptors than the corresponding normal cells (Firestone, 1994). Evidence for a high expression of LDL receptors on tumour cells was obtained by measuring binding and processing of LDL by tumour cells in culture. Additional evidence came from clinical studies in which a correlation was established between increased clearance of LDL from the circulation and tumour burden. Especially tumours of gynaecological origin and myeloid leukaemic cells, but also colon, kidney, lung and brain tumours, were found to express exceptionally high amounts of LDL receptors (Firestone, 1994). The high expression of LDL receptors on this variety of malignant cells makes LDL an attractive carrier for receptor-mediated delivery of anti-neoplastic drugs to tumours.

In a number of studies it was shown that lipophilic drug molecules could be incorporated in LDL particles without affecting the recognition of the particles by the LDL receptor. Furthermore, killing of cultured tumour cells was achieved with various drug–LDL complexes (Vitols *et al.*, 1985; Firestone *et al.*, 1984; Tokui *et al.*, 1994; Mosley *et al.*, 1981; Lundberg, 1993, Lestravel-Delattre *et al.*, 1992). However, to evaluate further the potential clinical application of LDL-mediated selective drug delivery to tumours *in vivo*, it is essential to develop an appropriate tumour-bearing animal model. In a few earlier studies, the association of radio-iodinated LDL with tumours was studied in tumour-bearing mice (Norata *et al.*, 1984; Lombardi *et al.*, 1989; Hynds *et al.*, 1984). Lombardi *et al.* (1989) studied a panel of seven tumours. They found that at 24 h after injection, the B16 melanoma tumour showed the highest tumour–liver ratio

Correspondence: MK Bijsterbosch
Received 11 January 1996; revised 21 March 1996; accepted 27 March 1996

(approximately 2). However, the use of directly iodinated LDL complicates the interpretation of the results, because the obtained values (at 24 h circulation) reflect only the amount of radiolabel present in the tissues at the time of sampling. The amount of radiolabel that is excreted from cells after degradation of the labelled LDL is not taken into account. Consequently, the amount of radioactivity present in the tumour and other tissues at the time of sampling does not reflect the total amount of LDL taken up during the experimental period. In the present study, loss of label from tissues after degradation of LDL is precluded by the use of tyramine cellobiose, a radiolabel that accumulates in cells after internalisation and lysosomal processing (Pittman *et al.*, 1983). It has been demonstrated in a number of studies that the radiolabelling with [$^{125}$I]tyramine cellobiose does not affect the recognition of LDL by the LDL receptor.

The aim of this study is to characterise further the receptor-mediated interaction of LDL with B16 murine melanoma cells. The expression of LDL receptors on the tumour cells is measured. Further, the processing of LDL by the cells is studied, as internalisation of drug-loaded LDL is essential for an effective therapy. The receptor-mediated uptake of LDL by the tumour *in vivo* is studied in B16 tumour-bearing mice. To provide an accurated determination of the total amount of LDL taken up *in vivo* by the tumour and non-tumour tissues, the LDL was labelled with [$^{125}$I]tyramine cellobiose, an intracellularly accumulating label.

### Materials and methods

### *Chemicals*

Sodium $^{125}$I, (carrier-free) was obtained from Amersham International, Amersham, UK. Dulbecco's modified Eagle medium (DMEM) was from Gibco BRL, Life Technologies, Gaithersburg, MD, USA. Fetal calf serum (FCS) was obtained from Hyclone Laboratories, Logan, UT, USA. L-Glutamine was from Merck, Darmstadt, Germany. A solution containing 50 000 IU penicillin and 50 mg ml$^{-1}$ streptomycin was obtained from Boehringer Mannheim, Mannheim, Germany. A solution of 2.5% (w/v) trypsin in Hanks' balanced salt solution without $Ca^{2+}$ and $Mg^{2+}$ was purchased from Flow Laboratories, Irvine, UK. All other chemicals were of analytical grade.

### *Isolation of lipoproteins*

Human LDL (density 1.024–1.063 g ml$^{-1}$) and high-density lipoprotein (HDL) (density 1.063–1.210 g ml$^{-1}$) were isolated from the serum of healthy fasted volunteers by density ultracentrifugation according to Redgrave *et al.* (1975). HDL was subsequently depleted of apoprotein E-containing particles, using a Sepharose–heparin column (Weisgraber and Mahley, 1980). The LDL and HDL preparations were dialysed against phosphate-buffered saline (PBS) (10 mM sodium phosphate buffer, pH 7.4, containing 0.15 M sodium chloride) and 1 mM EDTA, and sterilised by filtration through a 0.22 $\mu$m filter (Millipore, Molsheim, France). The concentration of the lipoprotein preparations was determined by measuring their apoproteins, according to the method of Lowry *et al.* (1951) with bovine serum albumin (BSA) as standard.

### *Reductive methylation of LDL*

LDL was reductively methylated as described by Weisgraber *et al.* (1978). An aliquot of 1 ml of solution of LDL (3–4 mg of apoprotein ml$^{-1}$) in 0.15 M sodium chloride containing 0.3 mM EDTA (pH 7.0) was mixed with 0.75 ml of 0.3 M sodium tetraborate (pH 9.0). To this mixture, which was kept on ice, were added 1 mg of sodium borohydride and 1 $\mu$l of formaldehyde. The reaction was allowed to react for a period of 30 min, during which 1 $\mu$l of formaldehyde was added every 6 min. The reaction was determined by separating, at 4°C, the methylated LDL from low molecular weight reactants using a Sephadex G-25 column (0.8 x 25 cm) eluted with 10 mM Tris-HCl containing 0.15 M sodium chloride (pH 7.4). The methylated LDL (MeLDL) fraction was subsequently dialysed at 4°C against PBS and 1 mM EDTA. A minimum of 80% of the lysine residues were modified by this procedure (determined as described by Habeeb, 1966).

### *Radioiodination of LDL and MeLDL*

For all *in vitro* experiments, LDL was labelled using the [$^{125}$I]iodine monochloride method as described in detail by Bilheimer *et al.* (1972). The specific radioactivity of $^{125}$I-labelled (Me)LDL was 100–200 d.p.m. ng$^{-1}$ of aproprotein, and less than 1% of the labelled material was trichloroacetic acid soluble.

For the *in vivo* experiments, MeLDL and LDL were labelled with [$^{125}$I]tyramine cellobiose ($^{125}$I-TC). Synthesis and subsequent radioiodination of TC was carried out as described earlier by Pittman *et al.* (1983). Coupling of [$^{125}$I]TC to (Me)LDL was done as follows. To 50 $\mu$l of 0.3 mM [$^{125}$I]TC were successively added 20 $\mu$l 0.75 mM cyanuric chloride in acetone and 10 $\mu$l of 3.0 mM sodium hydroxide. After 20 s, 20 $\mu$l of 2.25 mM acetic acid was added. The resulting activated ligand was added to 1–2 mg of (Me)LDL in 1 ml of 20 mM sodium tetraborate buffer, pH 9.0, that contained 0.12 M sodium chloride and 1 mM EDTA. After 30 min at room temperature, the reaction was quenched by the addition of an equal volume of 0.2 M ammonium biocarbonate. Unbound label was removed by exhaustive dialysis against PBS and 1 mM EDTA. Less than 1% of the labelled material was trichloroacetic acid soluble. The specific radioactivities of [$^{125}$I]TC-MeLDL and [$^{125}$I]TC-LDL varied between 10 and 50 d.p.m. ng$^{-1}$ aproprotein.

### *Culture of B16 melanoma cells*

The B16 (wild-type) melanoma cell line was obtained from Dr A Begg (The Netherlands Cancer Institute, Amsterdam, The Netherlands). The cells were cultured at 37°C in a humidified 5% carbon dioxide/air atmosphere in 125 cm$^2$ flasks (Costar) containing 5 ml of DMEM supplemented with 10% (v/v) heat-inactivated FCS, 2 mM L-glutamine, 50 IU penicillin ml$^{-1}$ and 50 $\mu$g ml$^{-1}$ streptomycin. Cells were subcultured twice a week by detaching the cells with trypsin (0.25% solution in $Ca^{2+}$- and $Mg^{2+}$-depleted Hanks' buffer), followed by renewal of medium the following day. Protein content per cell number was determined according to the method of Lowry *et al.* (1951) with BSA as standard.

### *Binding, association and degradation of [$^{125}$I]LDL by B16 cells in culture*

Cells were plated in 22 mm-diameter 12-well culture plates at a density of approximately 10 000 cells per well. Experiments were carried out 2 or 3 days later with subconfluently grown cells. Before the cell experiments, the culture medium was replaced by preincubation medium (medium with 1% (w/v) BSA instread of FCS). The cells were washed three times with preincubation medium (for 10, 10 and 30 min), and then cultured in this medium for a further 18 h. Experiments were started, after two quick washes with preincubation medium, by the addition of preincubation medium containing the indicated amounts of [$^{125}$I]LDL and the indicated additions.

*Binding* To determine binding of [$^{125}$I]LDL, the cells were incubated with the radiolabelled ligand and the indicated additives for 3 h at 4°C. After incubation, the culture plates were placed on ice. The cells were washed five times with ice-cold wash buffer (0.15 M sodium chloride, 2.5 mM calcium chloride and 50 mM Tris-HCl, pH 7.4) containing 0.2% (w/v)

BSA, followed by two washes with the same buffer without BSA. The cells were then lysed with 1 ml of 0.1 N sodium hydroxide, and the amounts of protein and radioactivity in the lysate were determined. The amount of cell protein was determined by the method of Lowry *et al.* (1951) with BSA as standard.

*Association and degradation* To determine association and degradation of [$^{125}$I]LDL the cells were incubated with the radiolabelled ligand and the indicated additives at 37°C. After the incubation, the culture plates were placed on ice and 0.5 ml of the medium was taken to measure the amounts of released degradation products of [$^{125}$I]LDL according to the method described earlier by van Berkel *et al.* (1981). The cell-associated radioactivity was determined as described above for cell-bound radioactivity.

*Regulation of receptor expression* For the experiments on the LDL receptor regulation on B16 cells, the cells were, after the initial 18 h of preincubation with preincubation medium (1% BSA), incubated for another 24 h with preincubation medium containing increasing amounts of LDL or HDL. Then the cells were washed at 37°C (for 10, 10 and 30 min) with preincubation medium without additions. The experiments were started by adding preincubation medium containing 10 $\mu$g ml$^{-1}$ [$^{125}$I]LDL. After 3 h, the plates were placed on ice and association and degradation were determined as described above.

*Tissue distribution of [$^{125}$I]tyramine cellobiose-labelled LDL and MeLDL in B16 tumour-bearing mice*

Male C57/B16 mice (10–15 weeks old) were used. The animals were kept on normal chow and had free access to water. Mice were inoculated subcutaneously on their back with $2 \times 10^5$ B16 cells obtained from cell culture. After development of the tumour to a just palpable size, the mice received an intravenous injection of radiolabelled LDL or MeLDL in the tail vein (dose of approximately 40 $\mu$g of apoprotein). In some experiments, mice were kept in metabolic cages for the collection of urine and faeces. Twenty-four h after the injection, a 0.4 ml blood sample was taken and the mice were sacrificed. Organs were removed by dissection, wiped with a tissue, weighed and the amount of radioactivity was determined. The radioactivity measured in a specific organ or tissue at the time of sampling was corrected for the amount of serum radioactivity present in the tissue. The amounts of serum in the organs and tissues were determined in seperate experiments using [$^{125}$I]BSA.

### Results

*Binding of LDL to B16 cells*

To determine the number of LDL receptors on B16 melanoma cells and the affinity of the receptors for LDL, cells were incubated at 4°C with increasing amounts of [$^{125}$I]LDL. After 3 h of incubation (i.e. at equilibrium conditions), the binding of [$^{125}$I]LDL to the cells was determined. Since receptors are not internalised at 4°C, only the binding of the ligand to cell surface receptors is measured. To determine aspecific binding of [$^{125}$I]LDL to the cells, an excess of unlabelled LDL was added. The specific receptor-mediated binding of [$^{125}$I]LDL was calculated by subtracting the non-specific binding from the total binding. From the results depicted in Figure 1, the dissociation constant ($K_d$) of the specific binding of LDL to B16 cells was calculated to be $5.9 \pm 2.3$ $\mu$g ml$^{-1}$ [i.e. 11 nM, assuming a molecular weight of 514 kDa for apoprotein B (Knott *et al*.,. 1986)]. The maximal binding of LDL to the cells was found to be $206 \pm 23$ ng of aproprotein per mg of cell protein. Taking into account the protein content of the B16 cells, it can be calculated from this result that about 220 000 LDL receptors are expressed per cell.

*Internalisation and catabolism of LDL by B16 cells*

To investigate the internalisation and processing of LDL by B16 cells, cells were incubated at 37°C for different time periods with 10 $\mu$g ml$^{-1}$ [$^{125}$I]LDL (Figure 2). A steady state in internalisation and degradation became evident after approximately 3–4 h. The maximal association was found to be $816 \pm 50$ ng of LDL mg$^{-1}$ of cell protein. The rate of degradation in the steady state was calculated to be $724 \pm 19$ ng of LDL mg$^{-1}$ of cell proliferation h$^{-1}$.

**Figure 1** Binding of [$^{125}$I]LDL to B16 cells. B16 cells were incubated at 4°C with the indicated amounts of [$^{125}$I]LDL. After 3 h of incubation, the total amount of cell-bound [$^{125}$I]LDL was determined (●). The aspecific binding of [$^{125}$I]LDL to the cells (○) was determined by including unlabelled LDL in the incubations (500 $\mu$g ml$^{-1}$). The receptor-specific binding (- - - -) was calculated by subtracting the aspecific binding from the total binding. Binding of [$^{125}$I]LDL is expressed as ng of apoprotein bound per mg of cell protein. Values are means ± s.e.m. of four separate experiments.

**Figure 2** Association and degradation of [$^{125}$I]LDL by B16 cells. B16 cells were incubated at 37°C with 10 $\mu$g ml$^{-1}$ [$^{125}$I]LDL. At the indicated times, the amount of cell-associated radioactivity (○) and the amounts of degradation products in the medium (●) were determined. Values are means ± s.e.m. of four separate experiments.

The selectivity of the association and degradation was investigated in a competition experiment (Figure 3). The cells were incubated at 37°C with [$^{125}$I]LDL in the presence of increasing amounts of unlabelled LDL or methylated LDL. Reductive methylation of only 20% or more of the lysine residues on the apoprotein B molecule prevents the receptor recognition of LDL, without changing other physical–chemical parameters of the particle (Weisgraber *et al.*, 1978). The results presented in Figure 3 clearly indicate that unlabelled LDL competes with [$^{125}$I]LDL for binding to the receptor. The cell association was decreased to less than 15% of the control value by the addition of 200 $\mu$g ml$^{-1}$ of unlabelled LDL. Methylated LDL, however, did not compete with the iodinated LDL, even at high concentrations.

Chloroquine and ammonium chloride inhibit the lysosomal degradation of internalised proteins by raising the pH inside lysosomes (Seglen *et al.*, 1979). Both compounds were used to establish the involvement of the lysosomes in the degradation of LDL by the B16 cells. The association of [$^{125}$I]LDL by B16 cells was relatively unaffected by the addition of the lysosomotropic compounds (Figure 4a). However, the degradation of [$^{125}$I]LDL could be completely inhibited by these agents (Figure 4b), indicating that the LDL is processed in B16 cells via the lysosomal route.

It has been shown earlier for a number of cell types that the binding of LDL to its receptor is strictly $Ca^{2+}$ dependent (Goldstein and Brown, 1977). Figure 5 shows that the receptor-mediated association and degradation of [$^{125}$I]LDL by B16 cells is also $Ca^{2+}$ dependent. Both association and degradation were inhibited when the incubation medium was virtually completely depleted of $Ca^{2+}$. Only 0.2 mM $Ca^{2+}$ was sufficient to accomplish association and degradation of the ligand.

*Regulation of the expression of LDL receptors on B16 cells*

To study the ability of the B16 cells to adapt to changes in the supply of LDL, the regulation of the expression of the LDL receptor on these cells was investigated. In these studies, the cells were preincubated with increasing amounts of LDL. After the preincubation period, the expression of LDL receptors on the cells was determined by measuring the association and degradation of [$^{125}$I]LDL. The results from this experiment, presented in Figure 6, clearly show that the expression of LDL receptors on the B16 cells is subject to regulation by LDL cholesterol. The preincubation with 300 $\mu$g ml$^{-1}$ of unlabelled LDL decreased the association and degradation of LDL to 32% and 24% of the control values respectively. Figure 6 also shows the effects of preincubation with HDL (high-density lipoprotein). It can be seen that preincubation with HDL does not decrease the expression of LDL receptors on B16 cells, but may even cause a slight increase.

*Uptake of LDL in B16 tumour-bearing mice*

To compare the uptake of LDL by B16 tumours with uptake of LDL by non-maligant tissues, B16 tumour-bearing C57/

**Figure 3** Effects of unlabelled (Me)LDL on the association of [$^{125}$I]LDL by B16 cells. B16 cells were incubated at 37°C with 10 $\mu$g ml$^{-1}$ [$^{125}$I]LDL in the presence of the indicated concentrations of unlabelled native LDL (●) or MeLDL (▲). After 3 h of incubation, the amount of cell-associated radioactivity was measured. The association of [$^{125}$I-]LDL is expressed as a percentage of the control values obtained in the absence of competitor. The 100% values for cell association were 2124 ± 383 ng of apoprotein mg$^{-1}$ of cell protein. Results are means ± s.e.m. of two separate experiments.

**Figure 4** Effects of chloroquine and ammonium chloride on the association and degradation of [$^{125}$I]LDL by B16 cells. B16 cells were incubated at 37°C with 10 $\mu$g ml$^{-1}$ [$^{125}$I]LDL in the presence of 0.1 mM chloroquine (■), 10 mM ammonium chloride (□), or without additives (●). At the indicated times, the amount of cell-associated radioactivity (**a**) and the amount of degradation products in the medium (**b**) were determined. To compensate for slow leakage of $NH_4^+$ from lysosomes during the incubation, ammonium chloride was added repeatedly every 30 min. The data represent the mean ± s.e.m. of two seperate experiments.

**Figure 5** Effects of $Ca^{2+}$ on the association and degradation of [$^{125}$I]LDL by B16 cells. B16 cells were incubated at 37°C with 10 μg ml$^{-1}$ [$^{125}$I]LDL. The $Ca^{2+}$ concentration in the medium (standard 1.8 mM) was varied by the addition of extra calcium chloride or by chelation with magnesium EGTA (to obtain negative values for [$Ca^{2+}$]). After 3 h of incubation, the amount of cell-associated radioactivity (○) and the amount of degradation products in the medium (●) were measured. Association and degradation are expressed as percentages of the association and degradation at 1.8 mM $Ca^{2+}$, which were 1674±238 and 1013±319 ng apoprotein per mg of cell protein respectively. Values are means±s.e.m. of three separate experiments.

B16 mice were intravenously injected with radiolabelled LDL. Reductive methylation of LDL provides a well-established tool for the determination of non-specific uptake of LDL (Weisgraber *et al.*, 1978; Mahley *et al.*, 1980). Therefore, to be able to distinguish between receptor-mediated and aspecific uptake of LDL, tumour-bearing mice were also injected with methylated LDL. In these experiments, LDL and MeLDL were both labelled with $^{125}$I-TC, a label that is retained in cells after uptake of its ligand (Pittman *et al.*, 1983). Tyramine cellobiose is an established radiolabel that is widely used in studies on the *in vivo* metabolism of lipoproteins. Pittman *et al.* (1983) showed, both *in vivo*, in rats and *in vitro* with cultured human fibroblasts, that there is no difference in recognition of $^{125}$I-TC-labelled LDL and directly iodinated LDL by the LDL receptor. Therefore, the *in vitro* results obtained with the directly iodinated LDL can be directly correlated to the *in vivo* data obtained with TC-labelled LDL.

At 24 h after injection of [$^{125}$I]TC-LDL, 15±3% of the injected dose was left in the plasma. [$^{125}$I]TC-MeLDL was cleared more slowly: at 24 h after injection 23±1% of the dose was still in the plasma. The uptake of LDL and MeLDL by the tumour and normal tissues, determined 24 h after injection, is shown in Figure 7. Tissue–serum ratios are expressed as the uptake of LDL per g of organ divided by the amount of LDL per g of serum (density: 1.05 g ml$^{-1}$). As has been found earlier in other species (Dietschy *et al.*, 1993), adrenals, liver and spleen showed the highest tissue–serum ratios after injection of [$^{125}$I]TC-LDL, i.e. 1.17±0.2, 0.82±0.08 and 0.69±0.07 respectively. The observed uptake by these tissues was largely receptor mediated, as is obvious from the lower tissue–serum ratio of [$^{125}$I]TC-MeLDL. The relatively high amount of label in the intestines is probably derived from the liver. It has been shown before in rats that after uptake of [$^{125}$I]TC-LDL by the liver part of the label is excreted via the bile duct into the intestine (Kleinherenbrink-Stins *et al.*, 1990). The uptake of [$^{125}$I]TC-LDL by the tumour was, with a tissue–serum ratio of 0.40±0.07, the highest of all the other tissues.

The observed uptake of labelled LDL by the tumour was approximately 2-fold lower than the hepatic uptake (tumour–liver ratio of 0.48±0.06). This is in contrast with the study by Lombardi *et al.* (1989), in which it was found

**Figure 6** Effects of preincubation of B16 cells with LDL or HDL on the LDL receptor expression. B16 cells were preincubated at 37°C with the indicated amounts of unlabelled LDL (●) or HDL (○). After 18 h of preincubation, the cells were washed three times (10, 10 and 30 min between washes) at 37°C with medium containing 1% BSA. This was followed by an incubation of the cells with 10 μg ml$^{-1}$ [$^{125}$I]LDL. After 3 h of incubation, the amount of cell-associated [$^{125}$I]LDL (**a**) and degradation products in the medium (**b**) were measured. Data shown represent LDL receptor-mediated association and degradation as they are corrected for non-specific association/degradation (determined by including 300 μg ml$^{-1}$ unlabelled LDL in the incubation). The results are expressed as percentages of association and degradation in controls (no lipoprotein present in the preincubation). Control values of association and degradation were 1120±68 and 231±9 ng of apoprotein per mg of cell protein respectively. The results are means±s.e.m. of two different experiments.

that uptake by the tumour was approximately two times higher than the uptake by the liver. To investigate whether the difference in results could be explained by the different labels used ($^{125}$I in the earlier study *vs* [$^{125}$I]TC in this study), we directly compared in B16 tumour-bearing mice the fate of LDL-labelled with either label. At 24 h after injection, the serum concentration [$^{125}$I]LDL and [$^{125}$I]TC-LDL were very similar: 11±2% and 15±3% of the dose respectively. The observed tissue–serum ratios were, however, strikingly

**Figure 7** Tissue distribution of [$^{125}$I]TC-LDL and [$^{125}$I]TC-MeLDL in mice inoculated with B16 cells. Male C57 black mice, bearing a B16 melanoma, were injected with approximately 40 $\mu$g of [$^{125}$I]TC-LDL (hatched) or [$^{125}$I]TC-MeLDL (solid) in the tail vein. After 24 h, a 0.4 ml blood sample was taken and the mice were sacrificed. Radioactivity was measured in the indicated organs and tissues. For the determination of organ and tissue uptake, the measured values were corrected for residual serum present in the organ and tissue samples. Results are the means ± s.e.m. of three animals.

**Figure 8** Tissue distribution of [$^{125}$I]TC-LDL and [$^{125}$I]LDL in mice inoculated with B16 cells. Male C57 black mice, bearing a B16 melanoma, were injected with approximately 40 $\mu$g of [$^{125}$I]TC-LDL (hatched) or [$^{125}$I]LDL (solid) in the tail vein. After 24 h, a 0.4 ml blood sample was taken and the mice were sacrificed. Radioactivity was measured in the indicated organs and tissues. For the determination of organ and tissue uptake, the measured values were corrected for residual serum present in the organ and tissue samples. Results are means ± s.e.m. of three animals for [$^{125}$I]TC-LDL and two animals for [$^{125}$I]LDL.

different (Figure 8). The amounts of label in tissues (in particular in the high-uptake tissues liver, spleen and adrenals) of mice injected with [$^{125}$I]LDL were much lower than in mice injected with [$^{125}$I]TC-LDL. These findings indicate that in mice injected with [$^{125}$I]LDL the label is rapidly procesed and excreted by the tissues. In mice injected with [$^{125}$I]LDL, the amount of label in the tumour was about twice the amount of label in the liver, which is in good agreement with the previously reported data.

### Discussion

A variety of tumour cell types express high numbers of LDL receptors, which makes LDL a very interesting carrier for the selective delivery of drugs to these tumour cells. In this study, we explored the feasibility of using the murine B16 melanoma cell line for the evaluation of the effectiveness of LDL-mediated tumour therapy. For this purpose, we characterised the binding and processing of LDL by B16 cells in culture and we studied the uptake of LDL by B16 tumours inoculated in mice.

We found that B16 cells in culture specifically bind LDL with high affinity ($K_d = 5.9\ \mu g\ ml^{-1}$). Since the B16 cells bound maximally 206 ng of LDL per mg cell protein, which corresponds to approximately 220 000 LDL receptors per cell, the affinity of binding of LDL to the B16 cells is very similar to the affinity of binding of LDL to human parenchymal cells ($K_d = 5\ \mu g\ ml^{-1}$; Kamps *et al.*, 1991). However, the human parenchymal cells bound under similar experimental conditions much less LDL (75 $\mu g\ ml^{-1}$ of cell protein). The requirements for the interaction of LDL with the receptors on the B16 cells are very similar to those of the 'classical' LDL receptors described by Brown and Goldstein, (1977). The lysine residues of LDL were found to be essential for the interaction with the receptor, as methylation of LDL competely abolished the capacity of the particle to compete for binding to the receptor. Further, the receptor-mediated association and degradation of LDL were $Ca^{2+}$-dependent.

For an efficient intracellular delivery of drugs by an LDL carrier, it is essential that the carrier is not only recognised, but also activity internalised by the cells. B16 cells were found to internalise and degrade LDL efficiently. The degradation of LDL by B16 cells could be completely inhibited by the addition of chloroquine or ammonium chloride to the culture medium. These findings indicate that LDL is internalised by B16 cells and is subsequently degraded in the lysosomes. Assuming a drug load of 100 molecules per LDL particle, a cellular protein content of 15% and a molecular weight of 514 kDa for apoprotein B, it can be calculated from the rate constants in the steady state, that a drug–LDL complex can deliver 21 $\mu$mol of drug per litre of cell volume per hour to the B16 cells. Since cytostatics, like for instance anthracyclines, are effective at an intracellular concentration in the $\mu$M range (Speth *et al.*, 1988), this clearly allows the administration of an effective cytotoxic dose.

The expression of LDL receptors on non-malignant cells is known to be regulated by the cells' need for cholesterol. We found that in B16 cells the expression of LDL receptors is also subject to regulation. Receptor-mediated association and degradation of [$^{125}$I]LDL were reduced by approximately 70% after preincubation with 300 $\mu g\ ml^{-1}$ LDL. Preincubation with HDL, a protein that extracts cholesterol from cells (reverse cholesterol transport), did not decrease the level of LDL receptor expression. In fact, a slight up-regulation of the receptor was observed. A similar behaviour was found for rat hepatocytes and HepG2 cells, a hepatoma cell line (Salter *et al.*, 1987; Havekes *et al.*, 1986). In contrast, human fibroblast cells were found to be more sensitive to receptor regulation, since preincubation of fibroblasts with as little as 20 $\mu g\ ml^{-1}$ of LDL led to a 75% decrease in LDL binding (Brown and Goldstein, 1975), indicating a relatively autonomic behaviour of the tumour compared with fibroblasts.

To evaluate the uptake of LDL by the B16 tumour *in vivo*, B16 cells were implanted in C57/BL6 mice and the tissue distribution of intravenously injected [$^{125}$I]TC-LDL was studied. In agreement with earlier studies on LDL catabolism *in vivo* (Dietschy *et al.*, 1993; Moerlein *et al.*, 1988), the liver (maintaining the cholesterol homeostasis), the adrenals (production of steroid hormones), the spleen and the intestinal tract were the major sites of recovery of LDL. The lower uptake of [$^{125}$I]TC-MeLDL by the liver, spleen and adrenals indicates that the uptake of LDL by these organs is mainly LDL receptor-mediated. The accumulation of LDL in the intestinal tract after 24 h is probably a consequence of direct transport of a small part of the $^{125}$I-TC-labelled apoprotein B from the liver via the bile to the gut, as was reported earlier for $^{125}$I-TC-labelled LDL and asialofetuin injected into rats (Pittman *et al.*, 1983; Kleinherenbrink-Stins *et al.*, 1990). Of all other tissues, the tumour showed the highest uptake of LDL, and the uptake by the B16 tumour was mainly LDL receptor-mediated.

The observed ratio of uptake of [$^{125}$I]TC-LDL by the B16 tumour *vs* uptake by the liver was $0.48 \pm 0.06$. In previous studies, using [$^{125}$I]LDL, tumour–liver ratios of 1.5–2 were found (Lombardi *et al.*, 1989; Ponty *et al.*, 1993). We show here, by comparing directly the uptake of [$^{125}$I]TC-LDL and [$^{125}$I]LDL, that the difference between the present and earlier studies is likely to be caused by the different labels used. [$^{125}$I]TC is an accumulating radiolabel, i.e. minimal loss of label from the tissues occurs after internalisation and degradation of [$^{125}$I]TC-LDL. Degradation of internalised [$^{125}$I]LDL leads to a rapid loss of labelled degradation products from the tissues. Consequently, the amounts of label present in the tissue at the time of sampling are an underestimation of the total amount of LDL taken up, in particular if (as in the liver) the internalised [$^{125}$I]LDL is rapidly degraded and excreted. Still, the recognition by the LDL receptor is similar for both labels (Pittman *et al.*, 1983). It was found that for some reason, the $^{125}$I label is retained relatively longer in the tumour than in the liver resulting in an overestimation (1.5–2 against 0.48) of the tumour–liver ratio. It is thus clear that an accumulating radiolabel is needed in order to determine accurately uptake of LDL by (tumour) tissues. $^{99m}$Tc has also been used as an accumulating radiolabel to follow the fate of LDL in B16 tumour-bearing mice and other animals. However, $^{99m}$Tc does not accumulate as well as [$^{125}$I]TC (Ponty *et al.*, 1993; Hay *et al.*, 1991) and the label is not sufficiently stable associated with LDL [25% dissociation of label during 24 h of storage at room temperature (Vallabhajosula *et al.*, 1988)]. We therefore conclude that our present values give the best possible estimates of the activity of LDL receptor-mediated uptake of tumour cells *vs* non-tumour tissues.

Uptake of LDL by the B16 tumour in the mouse thus appears to be lower than uptake by the liver, spleen and adrenals. If LDL is to be used as a carrier for delivery of cytotoxic compounds to the tumour, precautions are necessary to protect these organs from irreversible tissue damage. Uptake by the liver can be decreased by a diet enriched in cholesterol and triglycerides. The dietary fat is delivered to the liver by chylomicron remnants, which are taken up via liver-specific remnant receptors (van Dijk *et al.*, 1991). It has been shown in a number of studies that the high influx of cholesterol and fatty acids results in a substantial, up to 90%, down-regulation of hepatic LDL receptors (Angelin *et al.*, 1983). The expression of LDL receptors on tumour cells, which lack remnant receptors, will be unaffected by the diet. Further, the expression of LDL receptors in the liver, but also in the spleen, can be down-regulated by the administration of bile salts, like cholic acid or taurocholate. Uptake of LDL by tumour cells was found to be unaffected by the treatment (Hynds *et al.*, 1984). The uptake of LDL by the adrenals was shown to be greatly reduced in rats and rabbits by the administration of corticosteroids like hydrocortisone or dexamethasone (Hynds *et al.*, 1984; Isaacsohn *et al.*, 1986). Thus, it is possible specifically to down-regulate LDL receptors on non-malignant cells, so that cytotoxic compounds can be selectively delivered to tumour cells by an LDL carrier.

In conclusion, we have shown that cultured murine B16 tumour cells bind and internalise LDL via specific LDL receptors. The rate of internalisation of LDL was found to be sufficiently high to allow a significant intracellular accumulation of drugs incorporated in LDL carriers. B16 tumours inoculated in mice take up substantial amounts of circulating LDL via their LDL receptors. Liver, spleen and adrenals also show a high uptake of LDL, but LDL receptors in these organs can be specifically down-regulated, without affecting the expression of LDL receptors on tumour cells. We conclude that B16 tumour-bearing mice constitute a good animal model to evaluate the feasibility of LDL-mediated tumour targeting.

### References

ANGELIN B, RAVIOLA CA, INNERERITY TL AND MAHLEY RW. (1983). Regulation of hepatic lipoprotein receptors in the dog; rapid regulation of apolipoprotein B,E receptors, but not of apolipoprotein E receptors, by intestinal lipoproteins and bile acids. *J. Clin. Invest.*, **71**, 816–831.

BILHEIMER DW, EISENBERG S AND LEVY RI. (1972). The metabolism of very low density lipoproteins. I: Preliminary *in vitro* and *in vivo* observations. *Biochim. Biophys. Acta*, **280**, 212–221.

BROWN MS AND GOLDSTEIN JL. (1975). Regulation of the activity of the low density lipoprotein receptor in human fibroblasts. *Cell*, **6**, 307–316.

COUNSELL RE AND POHLAND RC. (1982). Lipoproteins as potential site-specific delivery systems for diagnostic and therapeutic agents. *J. Med. Chem.*, **25**, 1115–1120.

DIETSCHY JM, TURLEY SD AND SPADY DK. (1993). Role of liver in the maintenance of cholesterol and low density lipoprotein homeostasis in different animal species, including humans. *J. Lipid Res.*, **34**, 1637–1659.

FAN D, BUCANA CD, O'BRIAN CA, ZWELLING LA, SEID C AND FIDLER IJ. (1990). Enhancement of murine tumor cell sensitivity to adriamycin by presentation of the drug in phosphatidylcholine phosphatidylserine liposomes. *Cancer Res.*, **50**, 3619–3626.

FIRESTONE RA. (1994). Low-density lipoprotein as a vehicle for targeting antitumor compounds to cancer cells. *Bioconjugate Chem.*, **5**, 105–113.

FIRESTONE RA, PISANO JM, FALCK JR, MCPHAUL MM AND KRIEGER M. (1984). Selective delivery of cytotoxic compounds to cells by the LDL pathway. *J. Med. Chem.*, **27**, 1037–1043.

GOLDSTEIN JL AND BROWN MS. (1977). The low-density lipoprotein pathway and its relation to atherosclerosis. *Annu. Rev. Biochem.*, **46**, 897–930.

HABEEB AFSA. (1966). Determination of free amino groups in proteins by trinitrobenzenesulfonic acid. *Anal. Biochem.*, **14**, 328–336.

HAVEKES LM, SCHOUTEN D, DE WIT ECM, COHEN LH, GRIFFIOEN M, VAN HINSBERGH WM AND PRINCEN HMG. (1986). Stimulation of the LDL receptor activity in the human hepatoma cell line Hep G2 by high-density serum fractions. *Biochim. Biophys. Acta*, **875**, 236–246.

HAY RV, FLEMING RM, RYAN JW, WILLIAMS KA, STARK VJ, LATHROP KA AND HARPER PV. (1991). Nuclear Imaging analysis of human low-density lipoprotein biodistribution in rabbits and monkeys. *J. Nucl. Med.*, **32**, 1239–1245.

HYNDS SA, WELSH J, STEWART JM, JACK A, SOUKOP M, MCARDLE CS, CALMAN KC, PACKARD CJ AND SHEPHERD J. (1984). Low-density lipoprotein metabolism in mice with soft tissue tumours. *Biochim. Biophys. Acta*, **795**, 589–595.

ISAACSOHN JL, LEES AM, LEES RS, STRAUSS HW, BARLAI-KOVACH M AND MOORE TJ. (1986). Adrenal imaging with technetium-99m-labelled low density lipoproteins. *Metabolism*, **35**, 364–366.

KAMPS JAAM, KUIPER J, KRUIJT JK AND VAN BERKEL ThJC. (1991). Complete down regulation of low density lipoprotein receptor activity in human liver parenchumal cells by $\beta$-very low density lipoprotein. *FEBS Lett.*, **287**, 34–38.

KLEINHERENBRINK-STINS MF, VAN DER BOOM J, BAKKEREN HF, ROHOLL PJM, BROUWER A, VAN BERKEL ThJC AND KNOOK DL. (1990). Light- and immunoelectron microscopic visualization of *in vivo* endocytosis of low density lipoprotein by hepatocytes and Kupffer cells in rat liver. *Lab. Invest.*, **63**, 73–86.

KNOTT TJ, PEASE RJ, POWELL LM, WALLIS SC, RALL SC, INNERARITY TL, BLACKHART B, TAYLOR WH, MARCEL Y, MILNE R, JOHNSON D, FULLER M, LUSIS AJ, MCCARTHY BJ, MAHLEY RM, LEVY WILSON B AND SCOTT J. (1986). Complete protein sequence and identification of structural domains of human apolipoprotein B. *Nature*, **323**, 734–738.

LESTRAVEL-DELATTRE S, MARTIN-NIZARD F, CLAVEY V, TESTARD P, FAVRE G, DOUALIN G, SQALLI HOUSSAINI H, BARD J-M, DURIEZ P, DELBART C, SOULA G, LESIEUR D, LESIEUR I, CAZIN J-C, CAZIN M AND FRUCHART J-C. (1992). Low-density lipoprotein for delivery of an acrylophenone antineoplastic molecule into malignant cells. *Cancer Res.*, **52**, 3629–3635.

LOMBARDI P, NORATA G, MAGGI FM, CANTI G, FRANCO P, NICOLIN A AND CATAPANA AL. (1989). Assimilation of LDL by experimental tumours in mice. *Biochim. Biophys. Acta*, **1003**, 301–306.

LOWRY OH, ROSEBROUGH NJ, FARR AL AND RANDALL RJ. (1951). Protein measurement with the Folin phenol reagent. *J. Biol. Chem.*, **93**, 265–275.

LUNDBERG BB. (1993). Evaluation of methods for complexing prednimustine to low density lipoprotein. *Int. J. Pharmaceut.*, **99**, 275–283.

MAHLEY RW, WEISGRABER KH, MELCHIOR GW, INNERARITY TL AND HOLCOMBE KS. (1980). Inhibition of receptor-mediated clearance of lysine and arginine-modified lipoproteins from the plasma of rats and monkeys. *Proc. Natl Acad. Sci. USA*, **77**, 225–229.

MOERLEIN SM, DALAL KB, EBBE SN, YANO Y AND BUDINGER TF. (1988). Residualizing and non-residualizing analogues of low-density lipoprotein as iodine-123 radiopharmaceuticals for imaging LDL catabolism. *Nucl. Med. Biol.*, **15**, 141–149.

MOSLEY ST, GOLDSTEIN JL, BROWN MS, FALCK JR AND ANDERSON RGW. (1981). Targeted killing of cultured cells by receptor-dependent photosensitization. *Proc. Natl Acad. Sci. USA*, **78**, 5717–5721.

NORATA G, CANTI G, RICCI L, NICOLIN, A, TREZZI E AND CATAPANO AL. (1984). *In vivo* assimilation of low density lipoproteins by a fibrosarcoma tumour line in mice. *Cancer Lett.*, **25**, 203–208.

PITTMAN RC, CAREW TE, GLASS CK, GREEN SR, TAYLOR CA AND ATTIE AD. (1983). A radioiodinated, intracellularly trapped ligand for determining the sites of plasma protein degradation *in vivo*. *Biochem. J.*, **212**, 791–800.

PONTY E, FAVRE G, BENANIBA R, BONEU A, LUCOT H, CARTON M AND SOULA G. (1993). Bio-distribution study of $^{99m}$Tc-labelled LDL in B16-melanoma-bearing mice. Visualization of a preferential uptake by the tumor. *Int. J. Cancer*, **54**, 411–417.

REDGRAVE TG, ROBERTS DCK AND WEST CE. (1975). Separation of plasma lipoproteins by density gradient ultracentrifugation. *Anal. Biochem.*, **65**, 42–49.

SALTER AM, BUGAUT M, SAXTON J, FISHER SC AND BRINDLEY DN. (1987). Effects of preincubation of primary monolayer cultures of rat hepatocytes with low- and high-density lipoproteins on the subsequent binding and metabolism of human low-density lipoprotein. *Biochem. J.*, **247**, 79–84.

SEGLEN PO, GRINDE B AND SOLHEIM AE. (1979). Inhibition of the lysosomal pathway of protein degradation in isolated rat hepatocytes by ammonia, methylamine, chloroquine and leupeptin. *Eur. J. Biochem.*, **95**, 215–225.

SPETH PAJ, VAN HOESEL QGCM AND HAANEN C. (1988). Clinical pharmacokinetics of doxorubicin. *Clin. Pharmacokin.*, **15**, 15–31.

TOKUI T, TOKUI Y, ISHIGAMI M, TANZAWA K, IKEDA T AND KOMAI T. (1994). Targeting of an antitumour agent, RS-1541 (palmitoyl-rhizoxin), via low-density lipoprotein receptor. *Int. J. Pharmaceut.*, **110**, 277–283.

TOMLINSON E. (1987). Theory and practice of site-specific drug delivery. *Adv. Drug Del. Rev.*, **1**, 87–198.

TRAIL PA, WILLNER D, LASCH SJ, HENDERSON AJ, HOFSTEAD S, CASAZZA AM, FIRESTONE RA, HELLSTRÖM I AND HELLSTRÖM KE. (1993). Cure of xenografted human carcinomas by BR96-doxorubicin immunoconjugates. *Science*, **261**, 212–215.

VALLABHAJOSULA S, PAIDI M, BADIMON JJ, LE N-A, GOLDSMITH SJ, FUSTER V AND GINSBERG HN. (1988). Radiotracers for low density lipoprotein biodistribution studies *in vivo*: technetium-99m low density lipoprotein versus radioiodinated low density lipoprotein preparations. *J. Nucl. Med.*, **29**, 1237–1245.

VAN BERKEL ThJC, KRUIJT JK, VAN GENTT AND VAN TOL A. (1981). Saturable high affinity binding, uptake and degradation of rat plasma lipoproteins by isolated parenchymal and non-parenchymal cells from rat liver. *Biochim. Biophys. Acta*, **665**, 22–33.

VAN DIJK MCM, ZIERE GJ, BOERS W, LINTHORST C, BIJSTERBOSCH MK AND VAN BERKEL ThJC. (1991). Recognition of chylomicron remnants and $\beta$-migrating very low density lipoproteins by the remnant receptor of parenchymal liver cells is distinct from the liver $\alpha_2$-macroglobulin recognition site. *Biochem. J.*, **279**, 863–870.

VITOLS SG, MASQUELIER M AND PETERSON CO. (1985). Selective uptake of a toxic lipophilic anthracycline derivative by the LDL receptor pathway in cultured fibroblasts. *J. Med. Chem.*, **28**, 451–454.

WEISGRABER KH AND MAHLEY RW. (1980). Subfractionation of human high density lipoproteins by heparin-Sepharose affinity chromatography. *J. Lipid Res.*, **21**, 316–325.

WEISGRABER KH, INNERARITY TL, AND MAHLEY RW. (1978). Role of the lysine residues of plasma lipoproteins in high affinity binding to cell surface receptors on human fibroblasts. *J. Biol. Chem.*, **24**, 9053 – 9062.